# Disentangling the attention network test: behavioral, event related potentials, and neural source analyses

**DOI:** 10.3389/fnhum.2014.00813

**Published:** 2014-10-13

**Authors:** Alejandro Galvao-Carmona, Javier J. González-Rosa, Antonio R. Hidalgo-Muñoz, Dolores Páramo, María L. Benítez, Guillermo Izquierdo, Manuel Vázquez-Marrufo

**Affiliations:** ^1^Psychophysiology Unit (Lab B508), Department of Experimental Psychology, Faculty of Psychology, University of SevilleSeville, Spain; ^2^Laboratory for Clinical Neuroscience, Centre of Biomedical Technology (CTB), Technical University of Madrid (UPM)Madrid, Spain; ^3^Multiple Sclerosis Unit, Virgen Macarena University HospitalSeville, Spain; ^4^Department of Physiotherapy, Faculty of Nursing, University of SevilleSeville, Spain

**Keywords:** attention, neural networks, ANT, CNV, source analysis, P300, alerting, orienting

## Abstract

**Background:** The study of the attentional system remains a challenge for current neuroscience. The “Attention Network Test” (ANT) was designed to study simultaneously three different attentional networks (alerting, orienting, and executive) based in subtraction of different experimental conditions. However, some studies recommend caution with these calculations due to the interactions between the attentional networks. In particular, it is highly relevant that several interpretations about attentional impairment have arisen from these calculations in diverse pathologies. Event related potentials (ERPs) and neural source analysis can be applied to disentangle the relationships between these attentional networks not specifically shown by behavioral measures.

**Results:** This study shows that there is a basic level of alerting (tonic alerting) in the no cue (NC) condition, represented by a slow negative trend in the ERP trace prior to the onset of the target stimuli. A progressive increase in the CNV amplitude related to the amount of information provided by the cue conditions is also shown. Neural source analysis reveals specific modulations of the CNV related to a task-related expectancy presented in the NC condition; a late modulation triggered by the central cue (CC) condition and probably representing a generic motor preparation; and an early and late modulation for spatial cue (SC) condition suggesting specific motor and sensory preactivation. Finally, the first component in the information processing of the target stimuli modulated by the interaction between orienting network and the executive system can be represented by N1.

**Conclusions:** The ANT is useful as a paradigm to study specific attentional mechanisms and their interactions. However, calculation of network effects is based in subtractions with non-comparable experimental conditions, as evidenced by the present data, which can induce misinterpretations in the study of the attentional capacity in human subjects.

## Introduction

The attentional system is one of the most studied in cognitive neuroscience. Several models have been established, the most accepted being that of Posner and Petersen (1990; recently updated, Petersen and Posner, 2012). To study simultaneously diverse attentional networks, Fan et al. (2002) developed a task (termed attention network test; ANT) based in the combination of the cuing paradigm developed by Posner (1978), and the flanker task proposed by Eriksen and Eriksen (1974).

The attentional networks that can be studied by the ANT are as follows (Fan et al., 2005; Petersen and Posner, 2012). First, a general preparatory state or the “arousal” level needed for rapid detection of expected stimulus is managed by the alerting network, and is associated with increased activity in the right frontal lobe and right parietal lobe. These regions receive noradrenergic projections (related to alertness) from the locus coeruleus. Second, the movement of the attentional focus is allowed by the orienting network. The brain areas involved are the posterior parietal cortex, the thalamic pulvinar nucleus, the superior colliculus and the frontal eye fields. The orienting network is associated with the cholinergic system. Last, the executive network is responsible for conflict resolution (stimulus or response), error detection and inhibitory control, which is associated with the activity of the Anterior Cingulate Cortex (ACC) and the lateral prefrontal cortex. These regions contain a large number of dopamine receptors, suggesting that the dopamine system is involved in the executive network. Some recent studies also include vigilance or tonic alertness as another mechanism involved in the ANT (Roca et al., 2011; Martella et al., 2014). From the original study of Fan et al. (2002), several others have described the anatomy and physiology of these attentional networks in greater detail (Westlye et al., 2011; Chica et al., 2012; Yin et al., 2012), as well as possible variables that can modulate any of these three attentional networks (Roberts et al., 2006; Ishigami and Klein, 2010; Knight and Mather, 2013). A considerable number of them have applied ANT to several pathologies: Alzheimer (Fuentes et al., 2010); Multiple sclerosis (Urbanek et al., 2010); Schizophrenia (Neuhaus et al., 2010a); Mild Cognitive Impairment (van Dam et al., 2013); etc., describing how different attention mechanisms are impaired in these pathologies.

Although different regions of the brain may be locations of these attentional networks, it is suggested that there is a certain degree of independence between them (Fernández-Duque and Posner, 1997; Fan et al., 2005), and others have demonstrated that interaction between networks does exist. Callejas et al. (2005), using a task with auditory cues and visual targets, showed that the alerting network interferes with the executive network (causing a worse performance). They found a benefit from the alerting network for the orienting network, and also a benefit of the orienting network to the executive system. Fuentes and Campoy (2008), by varying SOAs between cues and targets, showed that the alerting network enhanced the orienting function in specific SOAs. Fan et al. (2007) found that the ACC was involved in both alerting (as a response anticipation) and executive functions (as response conflict), suggesting a possible locus for their interaction. Collecting data from 15 studies (*N* = 1129 healthy subjects), some authors have found that the networks were not independent and suggest caution in the interpretation of the data in this test (Macleod et al., 2010). McConnell and Shore (2011), using correlation analysis between all the conditions, showed that interactions between networks were present and pointed out the difficulty of measuring attention function through subtraction of the experimental conditions.

One possible way to clarify the interactions between these attentional networks is through neurophysiological measures such as the Event-Related Potentials (ERPs). In this particular test (ANT), different components can show what is going on in information processing, particularly with the attentional networks.

The first component that could be relevant is the contingent negative variation (CNV) (Walter et al., 1964) that is present between the presentation of a warning cue (S1) and the onset of the target stimuli (S2). Because the first stimulus (S1) usually serves as a preparatory or “warning” signal for the second “imperative” stimulus (S2), usually requiring a motor response, it is assumed that the CNV represents neuronal activity necessary for sensorimotor integration, which is related to planning, intentionality or decisional processes (e.g., the execution of externally paced, voluntary movements) (Rohrbaugh and Gaillard, 1983; Brunia, 1993). The CNV is comprised of at least two components or windows in the CNV interval (Rohrbaugh et al., 1976), one early or initial phase related to the orienting, and another later or terminal related to the expectancy. The early phase of the CNV is evoked by S1 and has been linked to sensory processes associated with evaluating the information contained in the warning stimulus and characterizing orienting activity, whereas the late CNV is considered to reflect both motor and cognitive preparatory processes associated with an imperative stimulus (Rohrbaugh and Gaillard, 1983; Ruchkin et al., 1986; Brunia, 1993; van Boxtel and Brunia, 1994).

Fronto-parietal networks involved in the CNV period are related to the motor planning or the sensory preparation and their neural generators, as determined by fMRI or EEG source analysis (Corbetta and Shulman, 2002; Gómez et al., 2003, 2007; Peelen et al., 2004). In particular, Gómez et al. (2007) stated that the neural sources for the early phase of the CNV are placed in the SMA, whereas late phases are located in primary motor cortex (MI) and extraestriate regions (Gómez et al., 2003, 2007; Nagai et al., 2004).

Other studies have focused on the role of this component in temporal tasks. In explicit timing tasks, the supplementary motor area (SMA) is activated (Macar et al., 1999) and bilateral premotor cortex would be more involved in implicit timing tasks (Praamstra et al., 2006). However, the question of timing processes related to the CNV period remains unresolved (Coull, 2004; van Rijn et al., 2011).

Another question in the meaning of the CNV is its dependency on motor preparation. Thus, from a preparatory process viewpoint for voluntary movements, the relationship between the CNV and other slow cortical negative potentials (e.g., the readiness potential—RP) has in part been solved (Ikeda et al., 1997; Brunia et al., 2012), and there is also good evidence that the late CNV can be elicited in the absence of a motor response (Ruchkin et al., 1986; Brunia et al., 2012). In this respect, it is possible to disentangle an “expectancy-related cognitive mechanism” independent from a motor preparation for the S2 stimuli (Mento et al., 2013). The evidence indicates that the meaning of the CNV is still under debate, with several mechanisms being intertwined in the S1-S2 interval.

In the specific case of the CNV and its application in the ANT, few studies have been carried out and these have been mainly focused in pathological matters. Kratz et al. (2011), in analyzing the potential modulations in the CNV interval, found an increase of the amplitude in the late phase for the spatial cue (SC) condition with respect to the neutral cue condition in healthy controls. However, other cuing conditions were not analyzed. Missonnier et al. (2013), in comparing all the conditions, concluded that the CNV amplitude was related to the amount of information given for the cue. The CNV amplitude was larger for the more informative SC (alerting and orienting, timing and sensory benefit) compared to the central cue (CC) (alerting, timing benefit), and obviously so in no cue (NC) condition.

With regard to the ERPs belonging to the target stimuli, the modulations in the early ERPs caused by visual attention have described; for instance, when a target stimulus is presented in an attended location, its P1 and N1 components increase compared to the same stimulus displayed in an unattended location (Hillyard and Mangun, 1987; Posner and Dehaene, 1994). In the particular case of the previous presentation of a SC, valid cued targets had an increase in the P1 amplitude, indexing greater enhanced sensory processing (Wright et al., 1995; Gonzalez-Rosa et al., 2006). However, if invalid cues are used, there was an increase in the N1, indexing the need for reorientation of the attention resources (Wright et al., 1995). On the other hand, others have also consistently described an increase of the N1 component related to validly cued target stimuli (Hillyard and Anllo-Vento, 1998; Nobre et al., 2000).

In analyzing these components in the ANT, Neuhaus et al. (2010b) looked at the modulations of the N1 and found that the amplitude varied by the alerting and orienting networks. In particular, the lowest amplitude occurred for the NC condition, larger for the double cue condition, and at the highest for the SC condition.

Lastly, the P3 component has also been analyzed in relation to the congruence variable in the ANT performance. A very definite reduction in the amplitude of the P3 occurred for the incongruent compared to the congruent condition (Neuhaus et al., 2010a). Reduction of the P3 amplitude was related in this study to a higher level of difficulty, but with the need for an inhibition in the response for the incongruent condition. However, some alternative interpretations of the decrease in the P3 have been proposed. Kratz et al. (2011) mentioned that the lower amplitude for the P3 component in ADHD children in the ANT indicated less attentional resources for this group.

Despite all these previous studies, some questions remain unresolved about the ANT: (1) how is behavioral performance affected in this test when the SOA between the cue and target stimuli is extended to a fixed interval of 1 s? (2) Is there any modulation in the CNV trace by a tonic alertness in the NC condition? (3) Has it been confirmed that it is possible to observe different CNV amplitudes related to the availability of cuing information? (4) Which psychological mechanisms are indexed in the early and late phases of the CNV? (5) Which of the target stimuli ERP components is modulated by the interaction of attentional networks? (6) Does the P3 reflect, as in previous studies, a decrease in its amplitude caused by incongruent stimuli compared to the congruent? (7) Can neural source analysis identify specific and overlapped brain areas for the diverse cuing conditions prior to the onset of the target stimuli?

We hypothesized that fixed and longer SOA (1 s) between cue and target stimuli could probably benefit from the following factors: cue (central and spatial regard to the NC condition) and congruence (congruent vs. incongruent targets); however, it is probable that no interaction among these factors will be observed. A certain negative trend is also to be expected in the CNV for the NC condition prior to the onset of the stimuli related to a general tonic alertness present during the performance of an experiment. Moreover, more information provided by the cue will produce bigger amplitudes in the CNV. In particular, modulation in the amplitude for the CC condition will probably occur at the late phase of the CNV, associated with the timing process and general motor preparation. In the case of SC condition, a difference in amplitude compared to the NC and CC condition would be expected in early and late phases of the CNV, related to more specific motor and sensory preparation. With regard to the first locus for the interaction between networks, perhaps one of the early components (the P1 or the N1) or both could represent it. A decrease in amplitude in the incongruent condition for the P3 component will probably be found. Lastly, neural sources may show overlapping regions for all cuing conditions and some specific for each one.

## Materials and methods

### Ethic statement

This study was carried out in compliance with the Helsinki Declaration. All participants signed informed consents before their inclusion and the protocol was approved by the ethics committee of the University of Seville (project code: PSI2010-16825).

### Subjects

Twenty-five healthy adults (15 males, 10 females) aged from 22 to 58 years (mean 29.6 ± 8.7 years) took part in this experiment, only one being left-handed. All participants reported normal or corrected-to-normal visual acuity and had no history of neurological or psychiatric diseases.

### Stimuli and procedure

**Figure 1:**
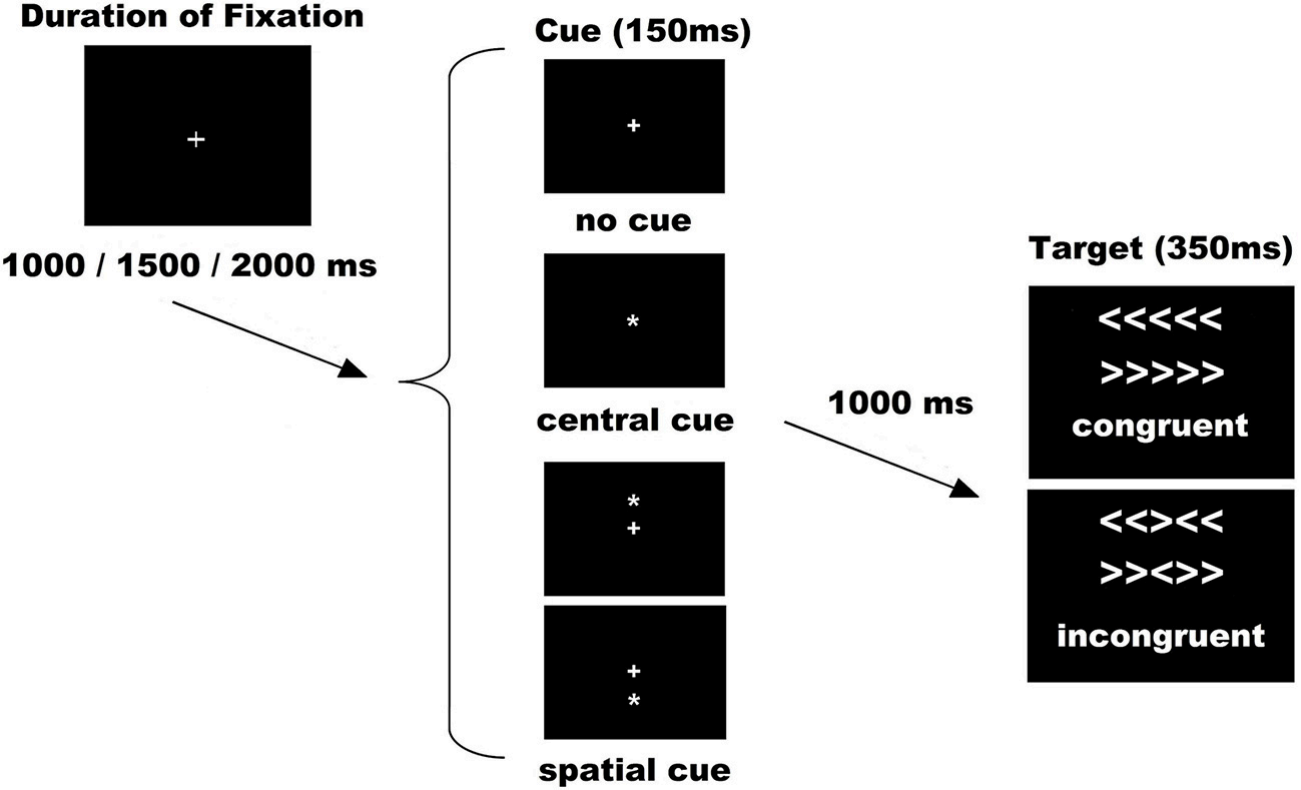
**Experimental procedure**. Possible combinations of sets of cues and targets were six: no cue congruent (NC-C), no cue incongruent (NC-I), central cue congruent (CC-C), central cue incongruent (CC-I), spatial cue congruent (SC-C), and spatial cue incongruent (SC-I). Abbreviation: ms, milliseconds.

The ANT was used as per the original authors (Fan et al., 2002). Stimuli consisted of a row of 5 horizontal white lines, with arrowheads pointing left or right, against a black background (see Figure 1). There were two types of target stimuli: a congruent target (C), when the central arrow was flanked by other arrows pointing in the same direction, and an incongruent target (I), when the flanking arrows pointed in opposite directions. Target stimuli represented a total visual angle of 3.28 on the x axis and 0.41 on the y axis. The congruent and incongruent trials occurred in equal proportions. Under each condition (congruent or incongruent), half were pointing to the left and half to the right. The subject's task was to indicate the direction of the central arrow by pressing the left button/arrow pointing to the left with the left thumb, or the right button/arrow pointing to the right with the right thumb. The target was presented in one of two locations, either 0.86 above or below the fixation cross in the center of the display, the cross appearing in the center of the visual display throughout the entire experiment. To engage the alerting and orienting processes, a cue (an asterisk symbol) was shown before the appearance of target. There were three cue conditions: NC, CC (at the fixation cross for alerting), and SC (at the target location for alerting plus orienting). All cues occurred in the same proportions. Cues were displayed with a visual angle of 0.41 on the x axis and 0.41 on the y axis. In the NC condition, a black square the same size as the cue was shown (not visible to the subjects) to adapt all the timings for the different cue conditions and make them comparable for ERPs analysis. As a result of the combination of target and cue conditions, the following six conditions were applied: no cue congruent (NC-C), no cue incongruent (NC-I), central cue congruent (CC-C), central cue incongruent (CC-I), spatial cue congruent (SC-C), and spatial cue incongruent (SC-I).

With respect to the duration of stimuli and interstimuli intervals, some adaptations were made to the original version (Fan et al., 2002; see Figure 1). The duration of the cue was 150 ms before a fixed duration of 1000 ms. The target (with flankers) was then presented for 350 ms. The time-window for participants' response was 1000 ms after target onset and the duration between the offset of the target and the start of the next trial was variable (1000, 1500, or 2000 ms). The experiment consisted of 288 trials in 2 blocks of 144. All the trials (diverse cues and different possible targets) were randomly presented in both blocks. With respect to behavior analysis, as suggested by others (Callejas et al., 2005; McConnell and Shore, 2011), we analyzed the interactions between conditions, but without subtractions (network effects) that could hide specific attentional mechanisms. Therefore, reaction time and accuracy were calculated for all conditions and averaged separately. Trials with an error were not included in the behavioral or ERPs analysis. All the participants were instructed to respond as quickly and accurately as possible.

### EEG procedure

**Figure 2:**
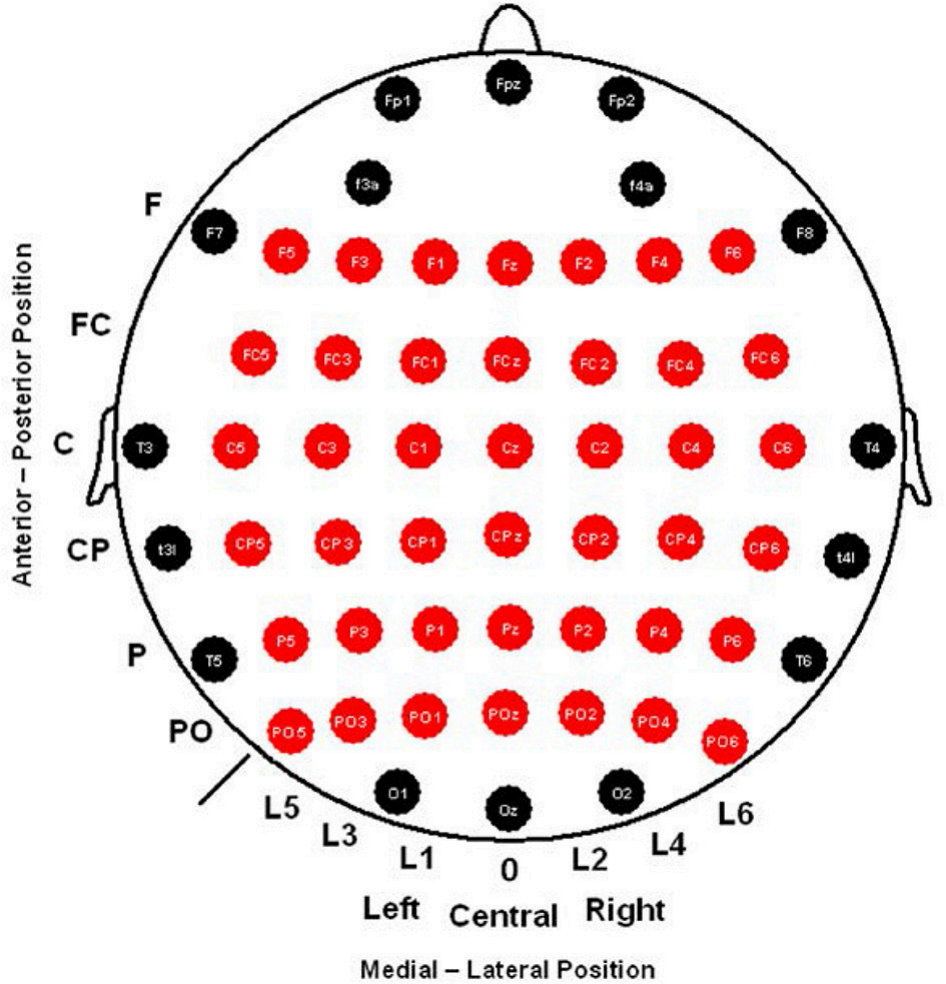
**The 58 scalp electrodes recorded and sets of 42 electrodes analyzed (in red) for ERPs (CNV, P1, N1, and P3) that were used**.

The electroencephalogram (EEG) was recorded from 58 scalp electrodes (Fp1, Fpz, Fp2, F3A, F4A, F7, F5, F3, F1, Fz, F2, F4, F6, F8, FC5, FC3, FC1, FCz, FC2, FC4, FC6, T3, C5, C3, C1, Cz, C2, C4, C6, T4, T3L, CP5, CP3, CP1, CPz, CP2, CP4, CP6, T4L, T5, P5, P3, P1, Pz, P2, P4, P6, T6, PO5, PO3, PO1, POz, PO2, PO4, PO6, O1, Oz, O2) (see Figure 2), all of which were compared to an averaged reference. Vertical and horizontal electro-oculograms (VEOG and HEOG) were recorded. The electrode signals were amplified with BrainAmp amplifiers and digitally stored using Brain Vision Recorder software (Brain Products GmbH, Germany). The EEG signal was digitized at 500 Hz and filtered through the amplifier using a band-pass of 0.01–100 Hz, with the impedance below 5 kΩ during the experiment. Trials with a HEOG signal outside the ±75 μV range were rejected. To obtain a good and balanced signal-to-noise ratio between conditions, all the individual averages also comprised >45 artifact-free trials (Polich, 1986; Duncan et al., 2009).

For the CNV amplitude analysis, baseline correction was done 150 ms prior to the onset of the cue stimuli. The CNV amplitude was analyzed for each cue condition in a time-window of 500 ms prior to the arrival of the target stimulus (specifically in 100 ms intervals, see Event Related Potentials Analysis Section for more details). After that, and because modulation found in the CNV under every cue condition, baseline correction was done 200 ms prior to the onset of the target stimuli for post-stimulus ERP component analysis. As suggested by Duncan et al. (2009), the latency and amplitude of the P1 and N1 components were measured as follows: finding the electrode with the maximum amplitude, identifying the latency of this peak and then exporting the amplitude value at that latency for the rest of the derivations included in the analysis. In the case of the P3 component, Pz electrode showed the maximum amplitude and had two peaks in some cases that were not recognizable in all subjects for latency analysis. Therefore, only amplitude analysis based in a range defined in the grand average (300–700 ms after the target onset) was set for this component in both target conditions (congruent and incongruent). Derivations used to analyze latencies and/or amplitudes for all these components are depicted in Figure 2.

### Neural sources analysis/source modeling

The spatiotemporal dynamics of cortical sources underlying the CNV were also analyzed. We estimated the foci of activations that were time-locked to the stimulus onset for each time-point for each cue condition and participant using the sLORETA (Standardized Low Resolution Electromagnetic Tomography) method implemented into Brainstorm software (http://neuroimage.usc.edu/brainstorm/). This method estimates cortical generator structures without a priori assumptions regarding the location and/or number of current sources (Pascual-Marqui, 2002; Pascual-Marqui et al., 2002). All computations were based on the 3-shell realistic head model (Fuchs et al., 1999), using default EEG sensor positions for each participant. A total of 6430 distributed current dipoles were estimated at a spatial resolution of 5 mm for each participant, whose locations (but not orientations) were constrained onto the cortical surface of the MNI-Colin27 brain template (Montreal Neurological Institute—MNI).

Relative activation values per subject and condition were normalized by calculating z-scores at each time-point relative to the baseline activity within the −150 to 0 ms window previous to the onset of the cue stimulus. These z-scores used to plot cortical maps and the absolute values of the z-scores were then averaged across the subjects. Source reconstructions were performed on the waves obtained from the grand-average of each condition and subject in 2 CNV intervals, −500 to −400 ms and −100 to 0 ms before the target stimulus, which showed previous ERPs differences between experimental conditions during the CNV period. To examine which cortical regions were significantly activated for task condition and each selected time-window of the CNV, cue-locked source activation values were inspected against the baseline using paired *t*-tests. To correct for multiple comparisons, we used the approach of controlling the false discovery rate, FDR (Benjamini and Yekutieli, 2001), with a threshold of *p*_(FDR)_ < 0.01. Sources were set at a threshold of 50% according to full-width at half-maximum (FWHM) criterion (Fuchs et al., 1999), and only areas (sources) fulfilling FWHM criteria with a minimum cluster size of 8 voxels were further considered. Anatomical localization of significant regions and Brodmann areas (BA) were first identified using MNI space by the MNI2TAL tool (http://imaging.mrc-cbu.cam.ac.uk/imaging/MniTalairach), and then confirmed by Talairach Client software (http://www.talairach.org), with correction to Talairach space (Brett et al., 2002).

### Statistical analysis of behavioral data

For behavioral analysis (reaction time and accuracy), a Repeated Measures ANOVA (RM-ANOVA) was used with the following factors and levels: Cue factor (NC, CC, and SC) and Congruence factor (Congruent and Incongruent conditions).

### Event related potentials analysis

To analyze alerting and orienting attentional networks, the CNV amplitude was analyzed by a RM-ANOVA with the following factors and levels: Interval factor (5 levels, each level representing an interval of 100 ms previous to the onset of target stimuli); Cue factor (three levels, NC, CC, and SC); Anteroposterior location factor (6 levels, Frontal, Frontocentral, Central, Centro-parietal, Parietal and Parieto-occipital) and Medial-Lateral Position factor [7 levels, Line 5, Line 3, and Line 1 for the left hemisphere; Midline (o zero); Line 2, Line 4, and Line 6 for the right hemisphere] (see Figure 2).

Specifically for the NC condition, statistical significance of the difference between averaged ERP amplitude and zero value for each subject was calculated before the onset of the target stimuli. To do this, a *t*-test against zero was used in each of the 10 intervals of 100 ms prior to the onset of the target stimuli (fixed duration of the cue-target interval was 1 s). The level of significance was recalculated (*p* = 0.05/420 = 0.0001) considering that the number of comparisons, the number of electrodes × intervals, was 42 × 10 = 420.

To measure the modulations caused by the attentional networks in the amplitude of the P1 and N1 components, 2 RM-ANOVAs were used, one for each component analyzed using a 3 × 2 × 6 × 7 design: Cue factor (NC, CC, and SC), Congruence factor (congruent and incongruent targets), Anteroposterior location factor (Frontal, Frontocentral, Central, Centro-parietal, Parietal and Parieto-occipital) and Medial-Lateral Position factor [Line 5, Line 3, and Line 1 for the left hemisphere; Midline (o zero); and Line 2, Line 4, and Line 6 for the right hemisphere] (see Figure 2). The P1 and N1 latencies were analyzed using an RM-ANOVA for each component with a 3 × 2 design: Cue factor (NC, CC, and SC) and Congruence factor (congruent and incongruent targets).

Amplitude modulations of the P3 component were analyzed with a RM-ANOVA for the following factors: Congruence factor (congruent and incongruent targets), Anteroposterior location factor (Frontal, Frontocentral, Central, Centro-parietal, Parietal and Parieto-occipital) and Medial-Lateral Position factor [Line 5, Line 3, and Line 1 for the left hemisphere; Midline (o zero); and Line 2, Line 4, and Line 6 for the right hemisphere] (see Figure 2).

The variables were checked for normality using the Shapiro–Wilk test (*p* > 0.05). A Greenhouse-Geisser correction for sphericity was applied and *p* = 0.05 was considered significant. The Bonferroni correction was used in multiple comparisons *post-hoc* analysis.

## Results

### Behavioral data

**Figure 3:**
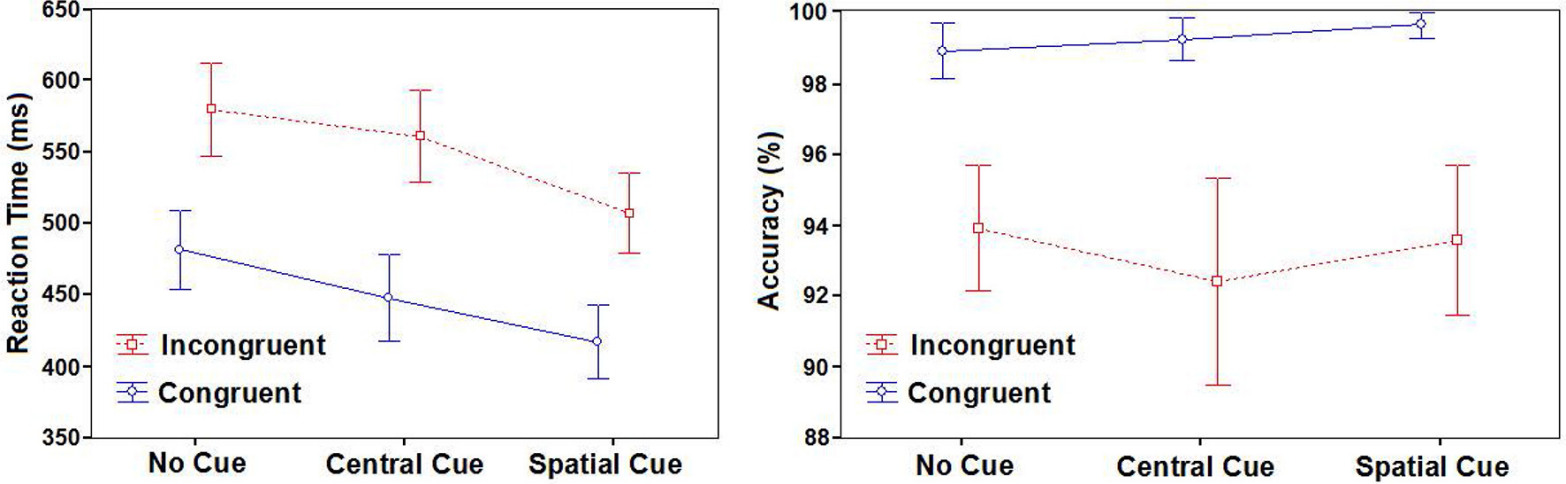
**Behavioral results**. Mean reaction times (in milliseconds) and accuracy (in percentages) with standard deviations according to cues and targets.

Mean Reaction Times (RTs) and Accuracy (Acc) for each experimental condition are summarized in Table 1 and Figure 3. A statistically significant result was found for the Cue factor [*F*_(2, 48)_ = 183.81; *p* < 0.001]. After *post-hoc* comparisons, the SC condition was faster than the CC (*p* < 0.001) and NC condition (*p* < 0.001), and for CC condition compared to the “NC” condition (*p* < 0.001). A statistical significant difference was also found with the Congruence factor [*F*_(1, 24)_ = 378.85; *p* < 0.001], because it had a faster congruent RT value than the incongruent one.

**Table 1:** **ANT behavioral results**.

**Conditions**	**RT (Mean ± *SD*)**	**Acc (Mean ± *SD*)**
NC-C	481 ± 67	98 ± 2
NC-I	579 ± 78	94 ± 4
CC-C	448 ± 73	99 ± 1
CC-I	560 ± 77	92 ± 7
SC-C	417 ± 61	99 ± 1
SC-I	507 ± 67	94 ± 5
Mean	498 ± 69	96 ± 2

Abbreviations: Acc, Accuracy (expressed in percentage); RT, Reaction Time (expressed in milliseconds); SD, standard deviation; NC-C, no cue congruent; NC-I, no cue incongruent; CC-C, central cue congruent; CC-I, central cue incongruent; SC-C, spatial cue congruent; and SC-I, spatial cue incongruent.

The interaction of Cue × Congruence factors was also significant [*F*_(2, 48)_ = 15.45; *p* < 0.001]. With *post-hoc* comparisons, all conditions were different in every possible comparison (all of them *p* < 0.001). The SC-C condition was the fastest, followed by the SC-I condition, the CC-C condition, and the CC-I condition in this order. Finally, the slowest conditions were the NC ones, with the NC incongruent condition being slower than the congruent one.

With respect to the accuracy measure, there was no effect of the Cue factor [*F*_(2, 48)_ = 0.84; *p* = 0.44] or the interaction of Cue × Congruence factors [*F*_(2, 48)_ = 1.42; *p* = 0.25]. Only the Congruence factor had a significant main effect [*F*_(1, 24)_ = 42.1; *p* < 0.001], with significantly higher values of accuracy for congruent targets than incongruent ones (*p* < 0.001).

### Event related potentials

#### The contingent negative variation

**Figure 4:**
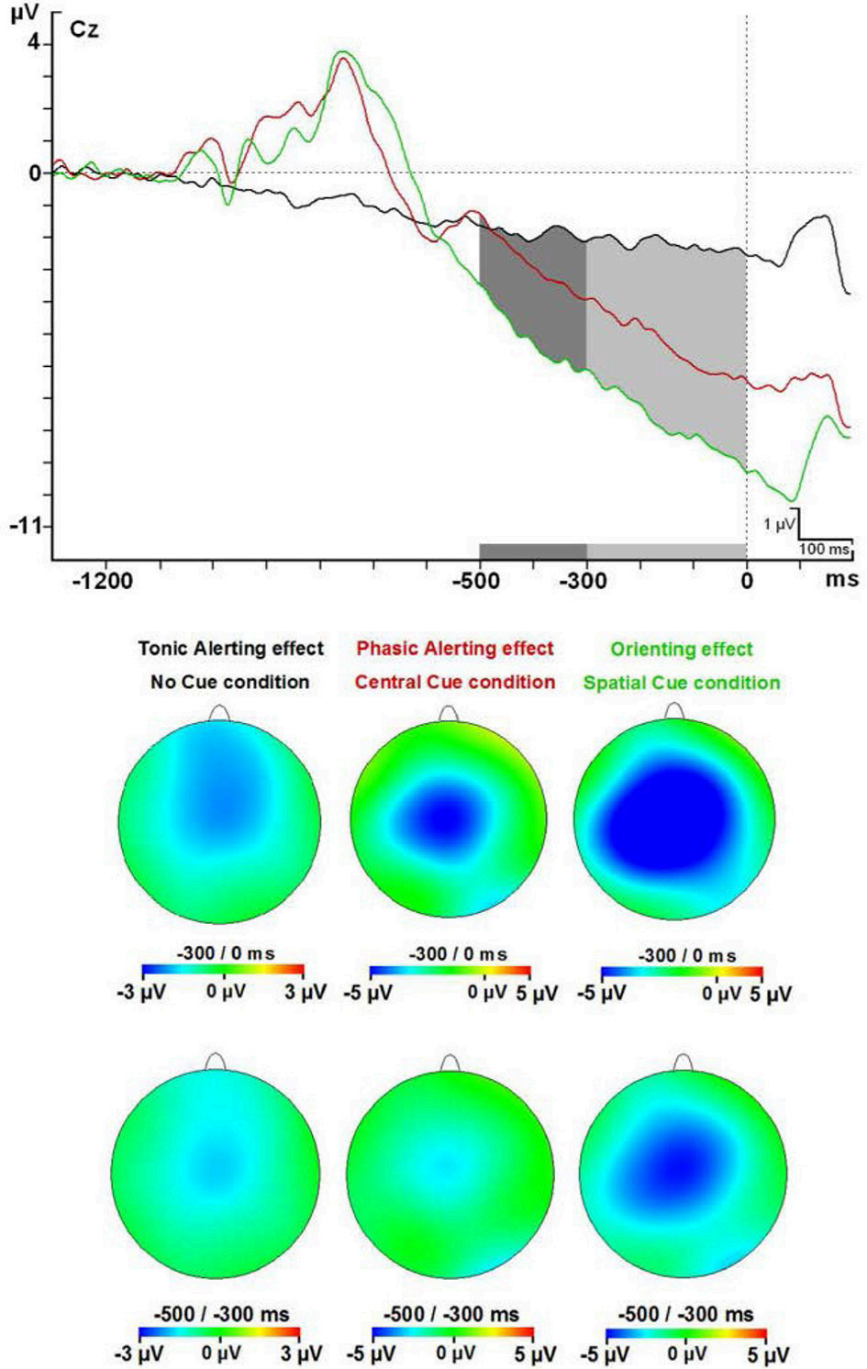
**Contingent negative variation modulations at Cz electrode and topographical maps**. Abbreviations: ms, milliseconds; μV, microvolts.

All the participants had the maximum amplitude value for the CNV in the FcZ or Cz derivations for every cue condition in each interval analyzed. Amplitude analysis of modulations in the CNV component gave statistical differences for diverse factors or interactions between them: Cue factor [*F*_(2, 48)_ = 16.62; *p* < 0.001], Cue and interval factors [*F*_(8, 192)_ = 17.87, *p* < 0.001], Interval, Cue, and Anteroposterior location factors [*F*_(40, 960)_ = 6.79; *p* < 0.001] and also Interval, Cue, and Medial-Lateral Position factors [*F*_(48, 1152)_ = 4.35; *p* < 0.001] (see Figure 4 and Table 2). *Post-hoc* analysis confirmed that the reasons of these significant effects were a higher amplitude (more negative) of the CNV for the SC condition than the NC and CC conditions in all intervals analyzed (500 ms previous to the onset of the target) (*p* < 0.05 for almost all electrodes analyzed, except Pz, POz, and PO2 electrodes in the −500 to −400 ms interval analyzed) (see Figures 2,4). The CC condition also had a higher negative amplitude value than the NC condition in the 300 ms before target onset (*p* < 0.05 for most electrodes analyzed, except F5, F3, F1, F2, F4, F6, FC5, FC3, FC6, C6, P5, P6, POz, and PO2 in the −300 to −200 ms interval; F5, F4, F6, FC3, FC6, and P3 electrodes in the −200 to −100 ms interval; and F5, F6, FC3, and FC6 electrodes in the −100 to 0 ms interval analyzed).

**Table 2:** **The CNV amplitude values**.

**Intervals**	**Mean ± *SD* (FCz)**	**Mean ± *SD* (Cz)**
**−100 to 0 ms**		
CNV-NC	−3.35 μ*V* ± 4.17	−2.20 μ*V* ± 2.27
CNV-CC	−5.98 μ*V* ± 5.68	−5.88 μ*V* ± 4.51
CNV-SC	−9.24 μ*V* ± 7.41	−8.40 μ*V* ± 4.86
**−200 to −100 ms**		
CNV-NC	−2.91 μ*V* ± 3.72	−2.00 μ*V* ± 4.17
CNV-CC	−4.95 μ*V* ± 4.63	−5.04 μ*V* ± 4.01
CNV-SC	−8.32 μ*V* ± 6.62	−7.62 μ*V* ± 4.81
**−300 to −200 ms**		
CNV-NC	−2.74 μ*V* ± 3.51	−1.97 μ*V* ± 1.85
CNV-CC	−3.95 μ*V* ± 3.72	−4.09 μ*V* ± 3.64
CNV-SC	−7.11 μ*V* ± 5.5	−6.39 μ*V* ± 4.4
**−400 to −300 ms**		
CNV-NC	−2.41 μ*V* ± 3.25	−1.76 μ*V* ± 1.89
CNV-CC	−3.22 μ*V* ± 3.08	−3.28 μ*V* ± 3.52
CNV-SC	−5.97 μ*V* ± 4.26	−5.65 μ*V* ± 4.33
**−500 to −400 ms**		
CNV-NC	−2.18 μ*V* ± 2.55	−1.77 μ*V* ± 1.59
CNV-CC	−1.95 μ*V* ± 2.93	−1.96 μ*V* ± 3.52
CNV-SC	−4.49 μ*V* ± 3.69	−4.15 μ*V* ± 4.60

Mean CNV (contingent negative variation) amplitude values of the electrode shown in each condition studied. Abbreviations: NC, no cue condition; CC, central cue condition; SC, spatial cue condition; SD, Standard deviation; μV, microvolts, Intervals are referred from the onset of the target.

**Figure 5:**
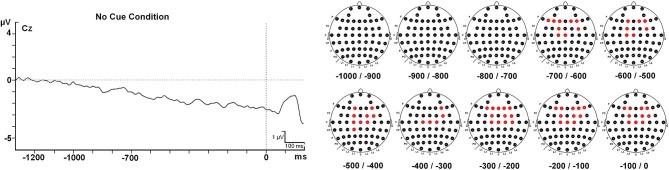
**CNV component modulation in the no cue condition and electrodes that showed statistical differences (in red) between averaged ERP amplitudes and zero value (*t*-test against zero) in each of the 10 intervals of 100 ms prior to the onset of the target stimulus analyzed**. Abbreviations: ms, milliseconds; μV, microvolts.

Importantly, a *t*-test against zero for the CNV in the NC condition showed that some electrodes in fronto-central areas gave statistically different results (*p* < 0.0001) compared to zero value for 7 intervals (700 ms before the target onset) (see Figure 5 for detailed locations of these effects). Negative amplitude values were found for the CNV in this condition at all intervals (see Table 3).

**Table 3:** **Amplitude values of the CNV in the no cue condition**.

	**Mean (−1000/−900)**	**Mean (−900/−800)**	**Mean (−800/−700)**	**Mean (−700/−600)**	**Mean (−600/−500)**	**Mean (−500/−400)**	**Mean (−400/−300)**	**Mean (−300/−200)**	**Mean (−200/−100)**	**Mean (−100/0)**
F5	n.s	n.s	n.s	−0.73	n.s.	n.s.	n.s.	n.s.	n.s.	n.s.
F3	n.s	n.s	n.s	−0.94	n.s.	n.s.	n.s.	−1.50	−1.45	−1.71
F1	n.s	n.s	n.s	−1.16	−1.35	−1.43	n.s.	−1.81	−1.86	−2.19
Fz	n.s	n.s	n.s	n.s.	n.s.	n.s.	n.s.	−1.63	n.s.	n.s.
F2	n.s	n.s	n.s	−1.05	−1.13	n.s.	n.s.	−1.66	−1.74	−1.97
F4	n.s	n.s	n.s	−1.11	−1.17	−1.26	−1.37	−1.61	−1.73	−1.93
F6	n.s	n.s	n.s	n.s.	n.s.	n.s.	n.s	n.s	−1.11	n.s.
FC1	n.s	n.s	n.s	−1.10	−1.27	−1.39	n.s	−1.75	−1.77	−2.04
FCz	n.s	n.s	n.s	n.s.	n.s.	n.s.	n.s	n.s	n.s.	n.s.
FC2	n.s	n.s	n.s	n.s.	n.s.	−1.29	n.s	−1.65	−1.74	−1.95
FC4	n.s	n.s	n.s	−1.05	−1.14	−1.32	−1.41	−1.71	−1.83	−1.99
C1	n.s	n.s	n.s	−0.94	−1.15	−1.41	−1.37	−1.62	−1.60	−1.72
Cz	n.s	n.s	n.s	−1.21	−1.40	−1.77	−1.76	−1.97	−2.00	−2.20
C2	n.s	n.s	n.s	n.s.	n.s.	−1.43	−1.45	−1.69	−1.75	−1.93
C4	n.s	n.s	n.s	n.s.	n.s.	n.s.	n.s	−1.30	n.s.	n.s.

Mean significant CNV (contingent negative variation) amplitude values in the no cue condition. Parentheses express temporal intervals in milliseconds. Considering that the number of comparisons were the number of electrodes × intervals, the level of significance was set to p = 0.0001. Only electrodes with statistical significant results (p < 0.0001) are shown. Abbreviation: n.s., no statistically significant.

#### The target P1 and N1 components

**Figure 6:**
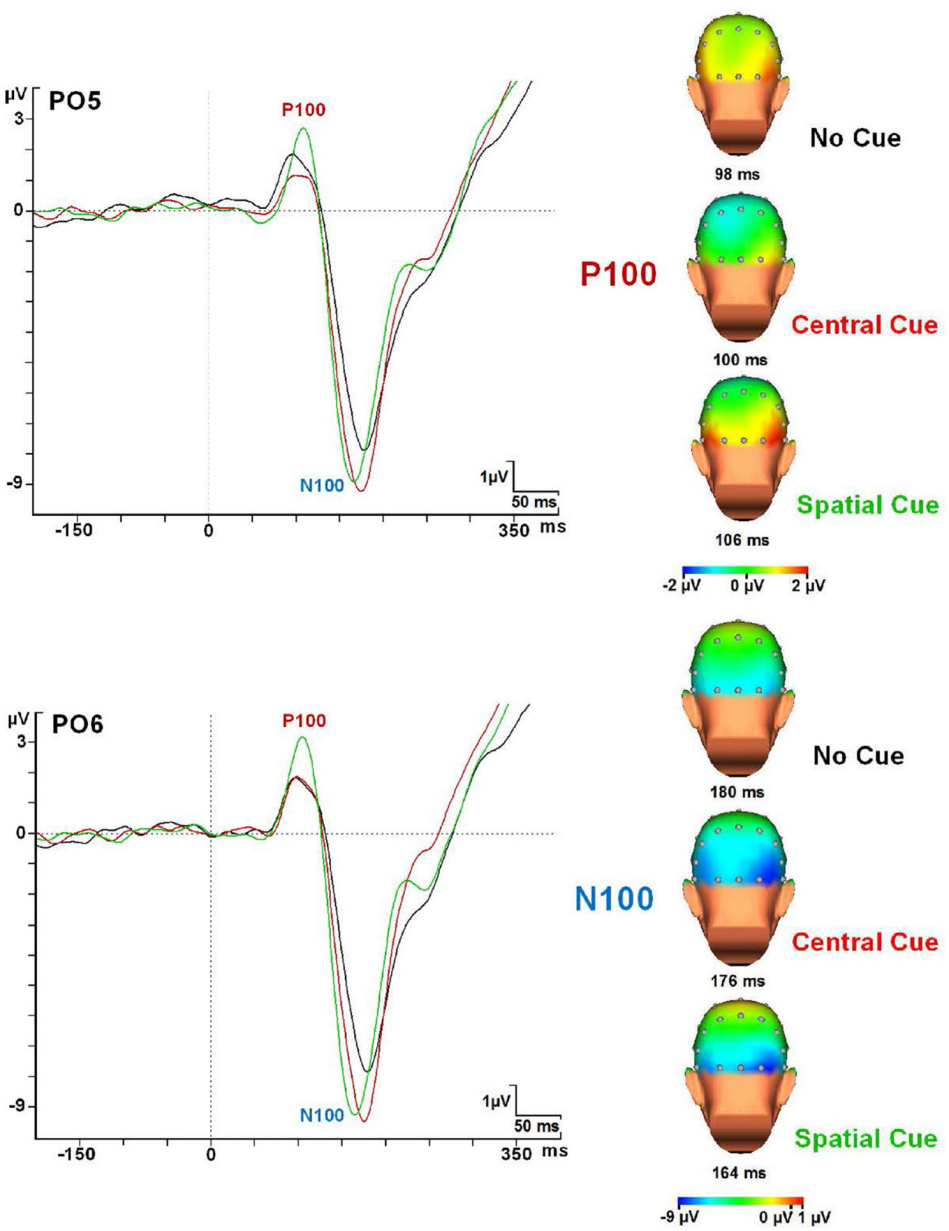
**P1 and N1 modulations in every cue condition at the PO5 and PO6 electrodes and topographical maps**. Abbreviations: ms, milliseconds; μV, microvolts.

PO5 and PO6 electrodes gave the maximum amplitude value for the P1 and N1 components for all cue × congruence conditions. Table 4 summarizes latency and amplitude values of the P1 and N1 component analyzed for each condition. RM-ANOVA showed no effect in the P1 latency for any factor. Concerning the amplitude of the P1 component (see Table 4), significant interactions of Cue × Anteroposterior location factors [*F*_(10, 240)_ = 6.7; *p* < 0.001] and Cue × Medial-Lateral Position factors [*F*_(12, 288)_ = 3.296; *p* < 0.001] were found. *Post-hoc t*-test indicated that the SC condition has the highest amplitude for the P1 compared to the CC and NC conditions in the parieto-occipital electrodes (*p* < 0.001 for PO5 and PO6 electrodes) (see Figure 6). NC and CC conditions were not significantly different with these electrodes (*p* > 0.05).

**Table 4:** **The P1 and N1 latency and amplitude values**.

			**NC-C (Mean ± *SD*)**	**NC-I (Mean ± *SD*)**	**CC-C (Mean ± *SD*)**	**CC-I (Mean ± *SD*)**	**SC-C (Mean ± *SD*)**	**SC-I (Mean ± *SD***
P1	Latency (ms)		114 ± 20	112 ± 28	111 ± 18	110 ± 21	113 ± 13	110 ± 16
	Amplitude (μV)	PO5	2.27 ± 2.95	2.30 ± 3.23	2.81 ± 2.41	1.80 ± 2.81	3.21 ± 4.32	3.63 ± 3.31
		PO6	3.16 ± 3.22	2.02 ± 3.31	3.59 ± 2.90	1.81 ± 2.47	4.28 ± 4.44	3.66 ± 4.34
N1	Latency (ms)		185 ± 18	183 ± 22	177 ± 11	177 ± 12	165 ± 12	170 ± 13
	Amplitude (μV)	PO5	−9.38 ± 6.20	−8.30 ± 4.62	−10.30 ± 5.41	−9.10 ± 4.82	−10.44 ± 5.66	−8.23 ± 3.89
		PO6	−8.60 ± 5.24	−8.61 ± 4.65	−9.77 ± 5.00	−9.81 ± 4.35	−10.79 ± 5.28	−9.24 ± 3.87

Amplitude values shown for P1 and N1 represent the mean value of the electrode shown in each condition analyzed. Abbreviations: SD, Standard deviation; NC-C, no cue congruent; NC-I, no cue incongruent; CC-C, central cue congruent; CC-I, central cue incongruent; SC-C, spatial cue congruent; and SC-I, spatial cue incongruent.

About the amplitude of the N1 (see Table 4), a significant effect of Cue factor [*F*_(2, 48)_ = 9.81; *p* < 0.001] and a significant interaction effect of Cue × Anteroposterior × Medial-Lateral Position factors [*F*_(60, 1440)_ = 2.428; *p* < 0.001] were observed. After *post-hoc* analysis, a higher amplitude was seen for the CC condition (*p* < 0.001) in the parieto-occipital scalp regions (PO5 and PO6 electrodes) compared to SC and NC conditions (see Figure 6).

**Figure 7:**
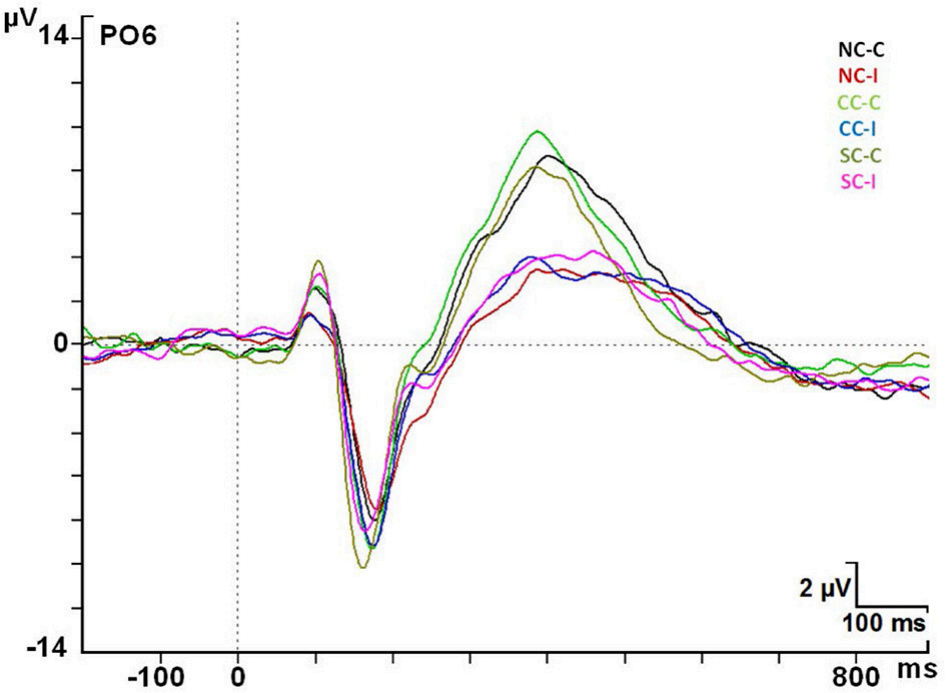
**ERP modulations at the PO6 electrode in every cue × congruence condition**. Abbreviations: ms, milliseconds; μV, microvolts.

With regard to the latency of the N1 component, RM-ANOVA showed an effect for the cue factor [*F*_(2, 48)_ = 17.65; *p* < 0.001]. Congruence factor or interactions of cue × congruence factors were not significant. However, *post-hoc* analysis showed that SC condition compared to CC condition gave faster values in the congruent target condition (*p* < 0.001), but no statistical differences were found between them in the incongruent target condition (*p* > 0.05). Moreover, *post-hoc* analysis also confirmed that the SC conditions gave faster N1 latency than the NC condition (both in the congruent and the incongruent target conditions) (*p* < 0.001). No statistical differences were found between NC and CC Conditions (*p* > 0.05) (see Figures 6, 7).

#### The target P3 component

The maximum amplitude for the P3 component occurred in the parietal regions (Pz) for target stimuli. There was a significant effect by Congruence factor [*F*_(1, 24)_ = 44.88; *p* < 0.001]. The Pz electrode gave mean values of 6.57 ± 3.35 μV for the congruent condition and 4.00 ± 2.42 μV for the incongruent one. *Post-hoc* analysis indicated a statistically significant increase of the amplitude for congruent targets compared to incongruent ones in the 42 electrodes analyzed (*p* < 0.05), except for the F1 electrode (*p* > 0.05) (see Figure 7).

#### Neural sources analysis

**Figure 8:**
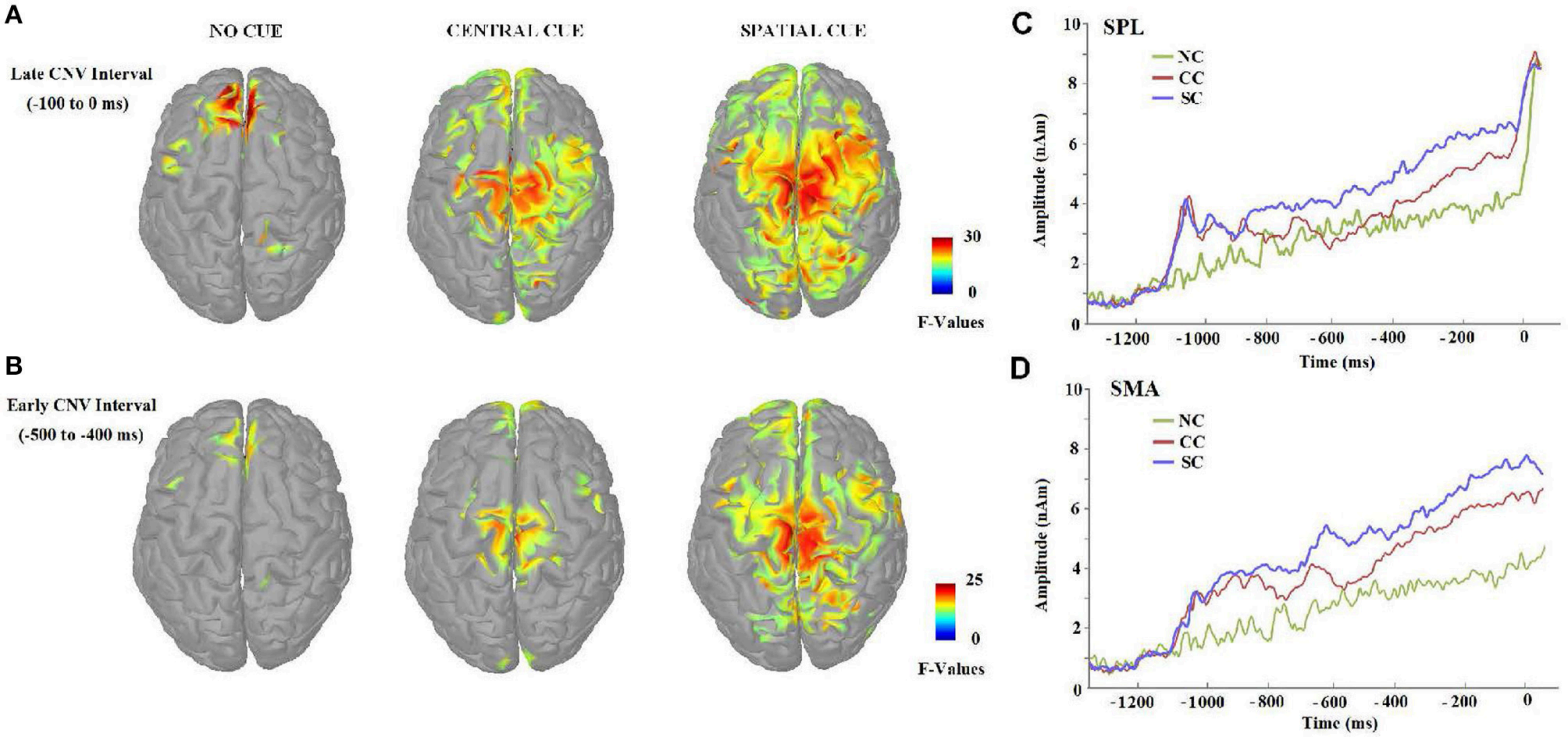
**(A,B)** Cortical activation maps presented in Z scores according to the baseline and showing significant activity (FDR-adjusted *p* < 0.01). Sources of the CNV effect were estimated in 2 CNV intervals of interest, −500 to −400 ms and −100 to 0 ms, before the target stimulus, which had shown previous ERP differences between experimental conditions during the CNV period. Source reconstructions maps were clipped at 50% (FWHM). **(C,D)** Cue-conditions effects on the mean time-course of activation in superior parietal lobe (SPL), and supplementary motor area (SMA) elicited during the ANT task for no cue (NC), central cue (CC), and spatial cue (SC) conditions in the 100 ms scoring windows at which statistical analyses were carried out. The data represent the grand-average source waveforms for all subjects, collapsing left and right hemispheres for the selected areas.

Figure 8 shows the distributed source activation pattern evoked by different ANT task conditions during the two selected CNV periods. BA, in which significant differences were found, are summarized in Tables 5, 6.

**Table 5:** **Summary of anatomical regions with significant activations compared to baseline in every cuing condition of the ANT test during the late CNV interval (−100 to 0 ms)**.

**Cue condition**	**ROIs**	**Brodmann area**	**Talairach coordinates**		
			***X***	***Y***	***Z***
**No cue**	*Frontal lobe*				
	L mFG	BA 6	−51	7	45
	L mFG	BA 8	−2	30	50
	L mFG	BA 9	0	34	33
	R mFG	BA 6	4	30	36
	R sFG	BA 6	14	23	57
	R preCG	BA 4	22	−22	71
	*Parietal lobe*				
	R PL	BA 5	14	−43	71
**Central cue**	*Frontal lobe*				
	L mFG	BA 6	−8	−17	72
	L mFG	BA 9	−43	10	37
	L mFG	BA 10	−4	57	0
	R mFG	BA 6	8	−17	72
	R mFG	BA 6	4	−9	48
	R mFG	BA 10	7	67	5
	L sFG	BA 6	−21	1	67
	L sFG	BA 8	−8	3	48
	R sFG	BA 6	1	3	70
	R sFG	BA 8	13	0	42
	R sFG	BA 10	5	45	−2
	L preCG	BA 6	−38	58	1
	*Parietal lobe*				
	R postCG	BA 5	3	−41	67
	R postCG	BA 30	17	−51	9
	R SPL	BA 7	17	−72	56
	*Occipital lobe*				
	L Cu	BA 18	−9	−97	22
	R Cu	BA 18	5	−90	17
	*Other regions*				
	L CG	BA 24	−2	−7	40
	L postCC	BA 30	12	−5	8
	L PCL	Gray BA 4	−	1	65
	L PCL	BA 31	−6	−37	45
	R PCL	BA 5	−5	−15	50
	R PCL	BA 5	6	−35	54
	R PCL	BA 31	6	−37	43
**Spatial cue**	*Frontal lobe*				
	L iFG	BA 9	−45	9	24
	L mFG	BA 6	−49	4	45
	L mFG	BA 6	−4	−2	50
	L mFG	BA 8	−22	19	43
	L mFG	BA 9	−46	10	36
	L mFG	BA 10	−2	61	13
	R mFG	BA 6	3	−10	58
	R mFG	BA 8	26	22	39
	R mFG	BA 10	3	56	8
	R mFG	BA 11	3	60	−12
	L sFG	BA 8	−5	19	49
	L sFG	BA 10	−2	63	−7
	L sFG	BA 11	−7	63	−13
	R preCG	BA 9	40	10	30
	*Parietal lobe*				
	R postCG	BA 5	6	−40	68
	L preCu	BA 7	−1	−67	44
	L preCu	BA 19	−23	−77	36
	L preCu	BA 31	−5	−48	36
	L preCu	BA 31	−13	−62	26
	R preCu	BA 7	3	−69	55
	R preCu	BA 31	3	−46	35
	R SPL	BA 7	31	−58	58
	*Occipital lobe*				
	L Cu	BA 18	−9	−98	20
	*Other regions*				
	L ACC	BA 32	−10	36	20
	R ACC	BA 32	7	40	12
	L CC	BA 23	−3	−14	29
	L postCC	BA 30	−15	−55	6
	L postCC	BA 31	−10	−45	44
	R postCC	BA 30	18	−51	10
	L PCL	BA 4	−8	−38	65
	L PCL	BA 31	−5	−24	45
	R PCL	BA 31	3	−12	46
	L Insula	BA 13	−38	−27	16
	R Insula	BA 13	41	−25	15
	L PHG	BA 19	−24	−45	−4

Analyses were thresholded at p (FDR-corrected) < 0.01 after FWHM criterion. Abbreviations: BA, Brodmann area; CC, cingulate cortex; CG, cingulate gyrus; Cu, Cuneus; L, Left; mFG, medial frontal gyrus; PCL, paracentral lobule; PHG, parahippocampal gyrus; PL, parietal lobe; preCG, precentral gyrus; PreCu, precuneus; postCG, postcentral gyrus; postCC, posterior cingulate; R, Right; sFG, superior frontal gyrus; SPL, superior parietal lobule; ROIs, regions of interest.

**Table 6:** **Summary of anatomical regions with significant activations compared to baseline in every cuing condition of the ANT test during the early CNV interval (−500 to −400 ms)**.

**Cue condition**	**ROIs**	**Brodmann area**	**Talairach coordinates**		
			***X***	***Y***	***Z***
**No cue**	*Frontal lobe*				
	L mFG	BA 6	−51	7	45
	R FEF	BA 8	4	37	41
	*Parietal lobe*				
	R PL	BA 7	15	−44	71
	*Other regions*				
	R CG	BA 32	4	21	40
**Central cue**	*Frontal lobe*				
	L mFG	BA 6	−3	−4	51
	R mFG	BA 6	1	−13	70
	R mFG	BA 10	6	48	−3
	L sFG	BA 6	−13	2	69
	L sFG	BA 10	−5	66	−10
	L sFG	BA 11	−12	59	−15
	R sFG	BA 6	5	−1	67
	R sFG	BA 10	15	66	12
	R sFG	Gray BA 11	9	65	−11
	R preCG	BA 4	12	−30	64
	*Parietal lobe*				
	L postCG	BA 3	−8	−33	61
	*Occipital lobe*				
	L Cu	BA 19	−9	−97	24
	R Cu	BA 18	8	−92	19
	*Other regions*				
	L CG	BA 24	−6	−4	49
	L PCL	BA 31	−4	−14	44
**Spatial cue**	*Frontal lobe*				
	L mFG	BA 6	0	−14	70
	L mFG	BA 6	−6	−12	70
	L mFG	BA 9	−43	9	35
	R mFG	BA 8	25	2	38
	R mFG	BA 9	38	11	28
	L sFG	BA 6	−8	2	69
	R sFG	BA 6	8	−2	69
	R preCG	BA 4	65	−4	21
	*Parietal lobe*				
	L preCu	BA 7	0	−59	50
	R preCu	BA 7	1	−68	41
	R SPL	BA 7	23	−58	67
	*Others regions*				
	L PCL	BA 31	−8	−17	45
	R PCL	BA 31	4	−2	45

Analyses were thresholded at p(FDR-corrected) < 0.01 after FWHM criterion. Abbreviations: BA, Brodmann area; CC, cingulate cortex; CG, cingulate gyrus; Cu, Cuneus; L, Left; mFG, medial frontal gyrus; PCL, paracentral lobule; PHG, parahippocampal gyrus; PL, parietal lobe; preCG, precentral gyrus; PreCu, precuneus; postCG, postcentral gyrus; postCC, posterior cingulate; R, Right; sFG, superior frontal gyrus; SPL, superior parietal lobule; ROIs, regions of interest.

During the first CNV time-window of interest (−500 to −400 ms before the target stimulus), source analysis showed the specific neural network activated when no warning cue was presented (NC) in comparison to when there was a cue preceding the target stimulus. Figure 8 also shows that a focal source of activity occurred within the frontal areas, confined to frontal eye fields (BA 8), with additional sources in the parietal and motor areas. CC led, indeed, to an increased bilateral activation of motor areas (BA 4/6) and in the frontal pole (BA 10), as well as other medial and superior frontal areas (BA 11/24). On the other hand, the presence of a valid SC was associated with a more widely distributed activation neural pattern in bilateral middle frontal gyrus (BA 8/9), parietal lobule (BA 7), and motor (BA 4/6/31) areas.

Concerning source localizations obtained during the late CNV time period (−100 to 0 ms before the target onset), the absence of warning cue (NC) showed once again a similar pattern: an increased activation pattern of bilateral medial frontal areas (BA 8), accompanied by a focal activation of the right parietal lobule (BA 4/5), and other middle frontal areas (BA 6/9). However, as expected, different motor and sensorial brain areas were most strongly activated when subjects were attending to some cue preceding the target stimulus. More specifically, early activation of the frontal pole and motor areas spread throughout a more bilateral and wide activation of the same brain areas following CC, with further increased activity within the posterior cingulate areas (BA 31), the right parietal cortex (BA 5/7) and the bilateral occipital regions (BA 18). Similarly to the active brain areas during CC, a widespread network of cortical sources were identified for the SC condition involving the bilateral motor and cingulate areas (BA 6, BA 24/30/31/32), frontal (BA 9, BA8, BA 11) and parietal cortex (BA 7), and increased recruitment of new brain areas as bilateral insula cortex (BA 13) and others involved in visual processing (BA 18/19).

## Discussion

### Behavioral data

The classical result in behavioral measures for the ANT was obtained. A faster response was found for the SC condition compared to the CC condition, and for both compared to the NC condition. This result reflects that cuing improves the performance in the responses, as described by the founders of this test (Fan et al., 2002, 2005). In particular, CC condition could be considered as a timing cue and the SC condition as a timing and SC that improve the performance in this task, due to the fixed SOA between the onset of the cue and the onset of the target. With respect to the executive system, a significant statistical difference was found for the congruence factor, indicating faster responses for the congruent compared to the incongruent targets as in previous studies (Fan et al., 2002, 2005). With regard to the accuracy variable, a significant difference was found only for the congruence factor, with a worse performance for the incongruent condition. This result guarantees that the effect in reaction time cannot be explained by a speed-accuracy trade-off (incongruent targets had slower responses and poorer accuracy values). With regard to the interactions between these two conditions, *post-hoc* tests showed that all cue × congruence comparisons were statistically different. Therefore, our data strictly show no interaction between cue and congruence conditions. These results suggest that, all conditions in our study represent a diverse set of cognitive mechanisms (probably some shared and others not) that account for reaction time differences. The data we obtained supports the notion that the timing between cue and target stimuli is relevant in determining the possible interactions of alerting, orienting and executive networks, as previously noted (Callejas et al., 2005; Fuentes and Campoy, 2008). In the vast majority of the ANT studies, SOAs between cue and target were quickly established (~400 ms); in our case, this interval was lapsed for 1 s, which maybe the cause in finding behavioral differences in all conditions and non-specific interactions between the attentional networks, as found by others (Fan et al., 2005; Fuentes and Campoy, 2008). Due to the differences found in the behavior, but the impossibility of disentangling the cognitive processes involved in the diverse conditions, neurophysiological indexes were analyzed.

### Event related potentials

#### Contingent negative variation

Statistical analysis showed that the CNV linked to the SC condition had a higher amplitude compared to the NC and CC conditions in the 500 ms prior to the onset of the target stimuli. Besides, the CNV from CC condition was greater in amplitude compared to the NC condition in the 300 ms prior to the onset of the target stimuli. An increase in the CNV amplitude caused by the presentation of a warning cue has already been described (Harter and Anllo-Vento, 1991; Wright et al., 1995; Gonzalez-Rosa et al., 2011). Moreover, a progressive increase in the CNV amplitude related to the amount of information contained in the cue occurred in our study, as suggested by others (Missonnier et al., 2013).

Importantly, the absence of the differences between the CNV from NC and CC conditions in the lapse of 500 to 300 ms prior to the onset of the target stimuli, and the statistical difference in the interval of 300 ms prior to the onset, suggest that the benefit from the CC (timing preparation) is specially located in the last phase of the CNV period. Previous studies have observed that the benefit in explicit timing tasks involved the SMA activation (Macar et al., 1999). Our neural source analysis showed that BA 6 (SMA) is activated in the CC condition as in the SC condition (both shared the timing benefit), but no effect was seen for the NC condition.

On the other hand, the difference between the SC condition and CC and NC conditions in the entire interval before the presentation of the target stimuli indicates that the benefit due to the certainty of the information provided by the SC probably involves several mechanisms occurring in different phases of the CNV. In particular, neural sources analysis showed that the SC condition caused activation in a higher number of areas compared to the CC and NC conditions. Among these areas, it was possible to find areas involved in decisional processes (BA 11 or BA 24, Orbitofrontal area or ventral anterior cingulate cortex), motor planning (BA 6, SMA) and sensory preparation (BA 19, associative visual cortex); see above for the relationship with the P1 component. The CC condition activates a similar set of areas (BA11, BA24, and BA31; Orbitofrontal area, ventral anterior cingulate cortex, and Dorsal Posterior cingulate cortex), but no significant activity for the BA19 or other visual areas, suggesting there was no specific sensory preparation.

Remarkably, both conditions (spatial and central) gave a different set of areas activated in the early and late phase of the CNV (Tables 5, 6), which suggests that, during the CNV interval, a complex organization in the brain activity takes place for both conditions sharing some mechanisms, but with some specific areas engaged for them individually. In summary, these findings are consistent with previous studies describing a general frontoparietal network that seems to be involved in the preparatory period between the cue (spatial and central) and the subsequent target (Corbetta and Shulman, 2002; Gómez et al., 2003, 2007; Fan et al., 2007); in the same way, it seems to point to a detailed spatio-temporal distribution of neural sources underlying the CNV, which would be related to different cue-dependent settings involved in specific cognitive preparation processes.

Apart from this result on the CNV for CC and SC conditions, a negative trend for the NC condition in the interval previous to the presentation of the target stimuli was found. During the 700 ms prior to the onset of the target, the CNV trace for the “NC” condition was statistically different from zero. This supports other studies where a better level of alertness (subjectively experienced) was related to an increased amplitude of the CNV (Ikemi, 1988; Linssen et al., 2011). It is surprising that a trend can be found when NC is presented in this period. At the same time, it is reasonable to think that a minimal level of general alertness oriented to the task is present in healthy subjects during an experiment. This modulation is not strictly related to the concept of vigilance or tonic alerting as has been studied by other groups adding an additional task to the ANT (Roca et al., 2011; Martella et al., 2014). It is related more to evidence found by Mento et al. (2013) where the execution of a cognitive task with fixed time parameters can build an expectancy that is echoed in the CNV. In the present paradigm, the subject awaits after the last target for the new stimuli that is coming (cue or target). After ~2 s, the subject knows that one of them will show up suddenly, and thus starts preparing for it. The interesting question in this case is that the construction of this task-related expectancy is created by the experience of the subject after some trials with all SOA possibilities, and where the maximum time between offset of the target and next stimuli is 2 s. This level of alertness (supposedly created by the subject estimations) is relevant in monitoring significant events during the performance of a task, and is impaired in subjects with ADHD (Spronk et al., 2008).

Analyzing the neural sources in the NC condition, it is possible to see that diverse areas are activated in different intervals of this trend. In the beginning, areas modulated are mainly related to the decisional processes (BA11, Orbitofrontal area) and monitoring (BA32, dorsal anterior cingulate cortex) which is logical, considering that the subject has to await both kind of stimuli (cues or targets). However, when the onset of a cue or a target is close (a timing built by subject experience), 2 specific areas are active (BA8 and BA10, frontal eye fields and anterior prefrontal cortex). Bunzeck et al. (2006) showed that when the demand of the task is reduced, activity of BA8 decreases. It seems reasonable that, when the subject is waiting for the cue or target, a higher demand is required and BA8 shows significant activity. With regard to the BA10, this area has been related to the maintenance of attention toward external stimuli (Burgess et al., 2007) and in the integration of the outcome of different cognitive operations for the achievement of goals in complex situations (Ramnani and Owen, 2004), as it is at that critical interval for a correct performance of the task.

#### The target P1 and N1 components

In the early ERPs for the target stimuli, the P1 showed a modulation in amplitude related to the cue factor. In particular, there was an increase in this component following a SC compared to NC or CC conditions. This is in accord with previous studies where higher amplitude has been related to the stimuli presented in the attended location (Hillyard and Mangun, 1987; Posner and Dehaene, 1994), which may be one reason for the improved behavioral performance. The sensory benefit is probably caused by preactivation during the CNV interval of the BA 19 in the SC condition. This area has been considered as a candidate for the P1 generator (Di Russo et al., 2002). No difference was found in the comparison between the P1 following NC or CC conditions, which reflects the lack of specific sensory preparation for both conditions. Differences in performance among them must be due to some other mechanisms (i.e., motor planning).

Regarding the latency of the N1 component, ANOVA showed a major effect caused by the cue factor, but no effects by the congruence factor or interaction between both factors. However, analyzed *post-hoc*, some interaction between these factors emerged. In particular, after a SC, the N1 showed a faster peak than when was preceded by a NC condition in both cases (target stimulus congruent or incongruent). However, comparison between SC and CC conditions showed that only with congruent targets was the N1 latency faster for the SC condition, with no statistical differences being found for the incongruent targets. The data suggest, in general, that the greater the information given by the cue, the lower is the latency of the N1 component. However, the interaction between the cue and congruence factors modulates in some way the latency of this component, possibly by showing that the executive system cannot use the facilitation of the SCs when an incongruent target is presented.

In the amplitude analysis of this component, there were not interactions between cue and congruence factors; however, some modulations were specifically produced separately by these factors. In the case of the cue factor, statistical analysis showed that the CC condition increases the amplitude of the subsequent N1 compared to the SCand NC conditions. It is reasonable to think that the increase in the CC compared to the SC condition is justified by the need in some cases of reorienting or moving the attentional focus to the target location, as previously suggested (Luck et al., 1990). However, the benefit of the attended location (SC condition), as shown by others (Luck et al., 1994), was not evident from our data. In this case, the reorientation effect in part could overcompensate for the increase in amplitude from the benefit of the spatial attention. However, due to the absence of any statistical difference between “NC” and SC conditions, it is more likely that the benefit of spatial attention does not occur in the course of the present data. This could be caused by the SOA we used for the CNV period being too long for the spatial attention benefit seen in previous by Luck et al. (1994). The lack of the visual attention effect in the N1 and the presence of modulation in the P1 reflect definite independence between these processes (Hillyard and Anllo-Vento, 1998; Hillyard et al., 1998).

On the other hand, another reason might explain the difference in the comparison between the CC and the NC conditions. McConnell and Shore (2011) suggested that the CC condition induces the subject to spread his attentional system in the visual field, whereas the other conditions (NC or SC) are more concentrated in specific regions (NC condition attending mainly to the fixation point, or the SC condition attending to a specific location defined by the cue). Two reports show that the N1 amplitude is increased for stimuli that require a larger sampling spread of the visual field for visual perception than for stimuli that require a small spread (Snyder et al., 2012; Benwell et al., 2014). Thus, our interpretation is that the modulation in the N1 component between CC and NC conditions reflects the need to readjust the global vs. local distribution of the attentional focus.

To sum up, different modulations in the N1 component reflect the way this component offers a complex set of modulations in the ANT and could be a specific neurophysiological index for the interaction of attentional networks, and consequently helpful to find its anatomical locations. Further research is necessary to confirm and interpret the role of this component in the interaction of attentional networks.

#### The target P3 component

Lastly, the amplitude of the P3 component was considerably increased for the congruent compared to the incongruent condition. This result has already been obtained using the ANT (Neuhaus et al., 2007, 2010a). Their conclusion was that the decrease for the P3 is related to response inhibition, but it was impossible to rule out the difficulty of the task involved as other have concluded (Polich, 1987; Hagen et al., 2006). However, not all the studies supporting the incongruent conditions have shown a lower less amplitude for this component. Zhang et al. (2013) found higher amplitude for the P3 component with incongruent compared to congruent stimuli. They believed that the decrease in the incongruent stimuli in the Neuhaus et al. (2010a) study is related to conflict at the response level, while the increase was possible because the conflict occurs at the perceptual level. Finally, it is also relevant that Rusnáková et al. (2011), using intracerebral recording, found that the P3-like potentials occurred more frequently with the incongruent than congruent stimuli in the temporal lateral neocortex and orbitofrontal cortex. These results show that the congruence variable comprises diverse cognitive mechanisms that make it difficult to reach clear conclusions. Indeed, another interpretation pointed to a decrement of the P3 amplitude related to a pathological (ADHD) reduction of attentional resources involved in the task (Kratz et al., 2011). However, a possible interpretation for this decrease could be the obverse. The P3 component, built by several mechanisms (evaluation of the stimuli, task relevancy, etc.), could show dispersion in time of these mechanisms when the difficulty is higher and greater synchronization of them when the demands of the task are lower. In the first case, greater attentional demands lead to lower amplitude in the P3 and vice-versa for the congruent condition, which is less demanding. In any case, to disentangle all these possibilities is beyond the aim this study and the precise meaning for the P3 component in this test needs further investigation.

### The problem of subtractions in the attention network test

All the evidence suggests that the different conditions that compose the ANT include different cognitive mechanisms, which are only shared in some cases. For instance, in the NC condition, the expectancy of the subject is quite different from the one that occurs with the CC and SC conditions. Essentially, in the former condition, the subject is naïve about the stimulus that will be displayed (cue or target), which makes it more difficult and slows the responses. In the CC and SC conditions, complete certainty of a target presentation is achieved and no new cue is expected. Therefore, the comparison between the NC and CC conditions is not merely a comparison between alert and not alert, as is usually suggested by studies using the ANT. The cognitive load between NC and CC/SC conditions is different, and more resources are needed in the former condition. Moreover, it is impossible to prepare any sensory or motor facilitation when a cue or a target is expected (it would be more a go-no go task rather than a choice task). This apart, the NC condition with regard to the CC condition is not even a valid comparison for alerting, since, as seen before, some negative trend in the CNV interval (representing a task-related expectancy) is seen in the NC condition so that it may not be engaged after the presentation of the CC.

In the case of the classical orienting network effect, subtraction between CC and SC conditions is not only based on two levels of orienting (oriented or not), but is also a different location that is attended (larger for the CC compared to the SC condition), and at the same time a higher number of elements (four possible targets in the CC condition with their corresponding possible motor responses rather than two for the SC condition). These differences cause a different initial state of the brain in processing the forthcoming target stimuli, which can induce different ways of processing the target stimuli thereby making it difficult to accept classical networks subtraction.

## Conclusion

The classical behavioral results of this test were observed—more informative cuing represents faster reaction times, and incongruent targets proved slower compared to the congruent ones. However, no specific interactions were found for all experimental conditions, probably due to the longer cue-target interval used in our experiments.

We have presented new findings regarding the ANT paradigm; specific modulations of the CNV are related to: a task-related expectancy presented in the NC condition; late modulation in the CNV triggered by the CC condition and representing probably a generic motor preparation; and an early and late modulation in the CNV for SC condition, representing more specific motor and sensory preactivations.

Regarding the ERPs for the target stimuli, the P1 amplitude increased following the SC suggesting an enhanced processing of spatially attended stimuli. More difficult to interpret have been the modulations for the N1 in latency and the amplitude that could be assigned to this component as the first locus for the interaction between attentional networks in the information processing of target stimuli. The P3 component has a bigger amplitude for the congruent than for the incongruent targets, which could represent different mechanisms of response inhibition, task difficulty, or simply an increase in the simultaneity of different processes indexed by this component.

Finally, neural sources show that, during the cue-target interval, some areas are shared between different cuing conditions, whereas others are specific to each one. Considering all these data, it seems reasonable that the network effects (calculated by subtractions) must be taken with caution, especially when clinical samples are considered and misinterpretations could be made. However, the ANT combined with ERPs is suitable for studying subtle mechanisms in information processing, including attentional ones as well as motor, decision-making, etc.

### Conflict of interest statement

The authors declare that the research was conducted in the absence of any commercial or financial relationships that could be construed as a potential conflict of interest.
